# Design, characterization and comparison of transdermal delivery of colchicine via borneol-chemically-modified and borneol-physically-modified ethosome

**DOI:** 10.1080/10717544.2018.1559258

**Published:** 2019-02-11

**Authors:** Yujia Zhang, Nan Zhang, Hui Song, He Li, Jin Wen, Xiaochuan Tan, Wensheng Zheng

**Affiliations:** a State Key Laboratory of Bioactive Substance and Function of Natural Medicines & Beijing Key laboratory of Drug Delivery Technology and Novel Formulation, Institute of Materia Medica, Chinese Academy of Medical Sciences & Peking Union Medical College, Beijing, China;; b Chinese Pharmaceutical Association, Beijing, China

**Keywords:** Transdermal delivery, gout, colchicine, borneol-chemically-modified ethosome, borneol-physically-modified ethosome

## Abstract

Gout is a kind of joint disease characterized by the accumulation of monosodium urate (MSU) crystals in the joint and its surrounding tissue, causing persistent hyperuricemia. Colchicine is the first choice of treatment for acute gout attacks. Due to strong toxicity of colchicines oral tablets, there are high fluctuations of blood drug concentration and serious irritation of gastrointestinal tract, and hence a transdermal preparation can help to slow down the blood drug concentration, reduce the frequency of drug taking, and improve the patients' compliance of the drug. The ethosome is a lipid carrier with high concentration of ethanol and has been proved to promote the penetration of drugs into the skin. Borneol (BO) is an excellent penetration enhancer in Chinese medicine, which can promote the entry of drugs into the skin. This paper prepared the borneol-physically-modified colchicine ethosome (COL-bpES) and used the prepared borneol-dioleoyl phosphoethanloamine (BO-DOPE) to prepare borneol-chemically-modified colchicine ethosome (COL-bcES). Compared to the free colchicine aqueous solution (free COL) and normal colchicine ethosome (COL-ES), the borneol-modified colchicine ethosome (COL-bES) demonstrated better drug penetration effect, while the particle size of the COL-bcES was lower than that of the COL-bpES. Toxicity, *in vitro* diffusion, pharmacokinetics and pharmacodynamics are superior to those of COL-bpES, providing a better delivery system for the treatment of small molecule inflammatory drugs.

## 1. Introduction

Transdermal delivery has some advantages over other delivery methods, such as avoidance of first-pass effect, better bioavailability and possible sustained-release effect. Hence, it has become one of the most popular research hotspots (Delgado-Charro & Guy, 2014). But due to the involvement of skin, which includes corium layer, epidermal layer, and stratum corneum, the range of drugs that penetrate the skin is very narrow. Only drugs with features such as molecular weight under 500 D, logP (n-Octanol/water partition constant) between 3 and 5 and melting point under 200 °C, can penetrate the skin effectively (Bhowmick & Sengodan, 2013). So, several ways of penetrating enhancements should be used to increase the field of penetrable drugs.

The frequently-used physical methods for penetration include microneedles, iontophoresis, etc. Chemical methods for penetration involve penetrating enhancers, nano-carriers, liposome, etc. The ability of traditional liposome carrying drugs to penetrate the skin remains unsatisfactory, and only uniquely-designed vesicles can achieve the transdermal goals (Cevc et al., 1998). Ethosome is a new vesicle which was first designed by an Israeli professor, E. Touitou in 1988 (Horwitz et al., 1999). Many researchers have showed that compared to traditional liposome, ethosome is an improved liposome with several advantages of easy creation method, higher stability, smaller particle size, higher penetrating effect, and no irritation, showing great research prospective in the area of transdermal drug delivery (Dayan & Touitou, 2000).

Borneol, a small liposoluble monoterpenoid molecule, has been reported to have many pharmacological effects, such as anti-inflammatory, antifungal, and antiviral. It can promote the distribution and absorption of drugs through biological barriers when used in combination with many kinds of Chinese medicines (Bhatia et al., 2008; Jiang et al., 2015). Also a lot of research on its mechanism of enhancing permeability is going on (Qi et al., 2013; Hou et al., 2017). At present, the mechanism of percolation of borneol is generally interpreted as: borneol can change the arrangement of the phospholipid molecules in the epithelial cells, change the close connection between the epithelial cells, open the cuticle, and increase the number and diameter of the endocytic vesicles with a reversible effect (Wang et al., 2017). Besides, borneol also inhibits the activity of P protein, which plays an important role in the entry of fat soluble drugs into cells, in the cell membrane, (Sherman et al., 2008).

Gout is a kind of joint disease characterized by the accumulation of monosodium urate (MSU) crystals in the joint and its surrounding tissue, causing persistent hyperuricemia (Ryszard et al., 2016). The acute gout attack is an acute inflammation caused by the disorder of purine metabolism and rising of hematuria acid by some influential factors (Pascual & Sivera, 2007). Severe pain and frequent attacks at night cause substantial adverse effects on patients’ lives. In the acute gout attack, the treatment mainly includes analgesic, and the commonly used drugs are nonsteroidal antiinflammatory drugs (NSAIDs), colchicine, glucocorticoids, and IL-1 antagonist. Colchicine is a classic drug used for the treatment of acute gouty arthritis in 1945. But colchicine remains more toxic in the treatment of acute gout attack, and its clinical adverse reactions mainly include gastrointestinal symptoms such as spastic abdominal pain, diarrhea, nausea, and vomiting. There is no effective antidote as the amount of poisoning is very close to the amount of treatment (Mehmood et al., 2016).

Hence, this study used colchicine (COL) as model drug, prepared borneol physically-modified colchicine ethosome (COL-bpES), and borneol chemically-modified colchicine ethosome (COL-bcES) by connecting borneol (BO) and dioleoyl phosphoethanolamine (DOPE) with succinic anhydride. Next, compared the effects of physically-modified and chemically-modified borneols through *in vitro* transdermal permeation, pharmacokinetics and pharmacodynamics, and evaluated the penetration promoting effects after modifying.

## 2. Materials and animals

Borneol (BO), stearic acid (SAA), 4-Dimethylaminopyridine (DMAP), N,N-Dimethylformamide (DMF), N-Hydroxysuccinimide (NHS), 1-Ethyl-3-(3-dimethylaminopropyl) carbodiimide (EDCI) were purchased from Aladdin Chemical Co. Lecithin was purchased from Lipoid GmbH. Egg yolk lecithin (PC-98T) and DOPE were purchased from A.V.T. (Shanghai) pharmaceutical Co., Ltd. Colchicine and scopolamine hydrobromide standards were purchased from the National Institutes for Food and Drug Control.

All experimental animals were purchased from Charles River Co., Ltd and relevant experiments were conducted with the approval of Peking Union Medical College & Chinese Academy of Medical Sciences Animal Care Committee.

## 3. Methods

### 3.1. Synthesis and characterization of BO-DOPE

BO-DOPE was synthesized by coupling borneol (BO) on the hydrophilic side of DOPE using succinic anhydride (SA) as a linker by esterification reaction and amidation. The detailed preparation process has already published in the International Journal of NanoMedicine (Song et al., 2018). The prepared BO-DOPE was dialyzed by using the dialysis bag with a specification of 500 Da for 24 h.

### 3.2. Preparation of three kinds of ESs

According to the total system mass of 6 g and 10% borneol modification rate, 12 mg borneol, 12 mg colchicine, 75 mg BO-DOPE, and 57 mg egg yolk were dissolved in 2.28 mL ethanol. Then 4.056 mL distilled water was injected into the lipid ethanol solution at 20 mL/h in closed conditions, and sonicated (250 W, 40 kHz) for 15 min at 25 °C to prepare borneol-chemically-modified colchicine ethosome (COL-bcES). 12 mg borneol, 12 mg colchicine, and 120 mg egg yolk lecithin were dissolved in 2.28 mL ethanol. After that, 4.08 mL distilled water was injected into the lipid ethanol solution at 20 mL/h in closed conditions, and then sonicated (250 W, 40 kHz) for 15 min at 25 °C to prepare borneol-physically-modified colchicine ethosome (COL-bpES). 12 mg colchicine, 120 mg egg yolk lecithin were dissolved in 2.28 mL ethanol. After that, 4.20 mL distilled water was injected into the lipid ethanol solution at 20 mL/h in closed conditions, and then sonicated (250 W, 40 kHz) for 15 min at 25 °C to prepare colchicine ethosome (COL-ES). The COL-bcES, COL-bpES and COL-ES were sonicated by ultrasonic probe (150 W, 50 Hz, ultrasonic 2 s, clearance 3 s) for 5 min and 0.22 µm filter membrane under liquid ice bath (Zhang et al., 2018).

### 3.3. HPLC analysis of the drug

The HPLC system consisted of a solvent delivery pump (2487, Waters Corporation, America) and an ultraviolet (UV) absorbance detector (2695, Waters Corporation). Chromatographic column consisted of Inertsil C-8 (250 mm × 3 mm, 3 μm); mobile phase of 45% methanol solution; detection wavelength of 254 nm; column temperature of 25 °C; velocity of 1 mL/min; and sampling amount of 20 μL. The linearity, specificity, intra- and interday variabilities and recovery of HPLC methods were also undertaken and satisfied the quantitative analysis request for the samples.

### 3.4. Characterization of three kinds of ESs

The particle size, polydispersity index (PdI), and zeta potential of the three kinds of ESs were measured by dynamic light scattering (DLS) method using a Zeta Potential/Particle Sizer (Malvern nano ZSP series) and diluted with 1:1 water. Morphological examination was performed using a transmission electron microscope (TEM), and samples for TEM were made by dropping 5 mg mL^−1^ aqueous sample on carbon-coated copper grids. The encapsulation efficiency was measured by ultrafiltration with ultrafiltration tube of 15 mL. 1 mL prepared ESs was added in the tubes, respectively, and centrifuged for 4 h under 5000 *g* (7000 rpm). The continuous filtrate was collected and diluted to 10 mL with distilled water, and then the concentration p1 was measured under the condition of “Section 3.3”. 100 μL COL-ES and 1 mL ethanol was added in 10 mL volume and sonicated for 30 min to demulsificate. The solution was diluted with distilled water to 10 mL, and the concentration p2 of colchicine was measured according to the chromatographic conditions under “Section 3.3”. The formula for calculating entrapment efficiency of ESs was: (p2*10 − p1*10)/(p2*10) * 100%.

### 3.5. Cytotoxicity assay

HaCaT was used to investigate the effects of COL-ES, COL-bpES, and COL-bcES on cell viability. The culture bottle was taken out of the incubator, the medium was removed, and 2 mL phosphate buffered saline (PBS, pH 7.2) was added to scrub the cells for two times to remove the medium. Add 1 mL trypsin, digest for 3 min, observe the cell shrinkage and roundness under microscope, and then add 2 mL medium to stop digestion. The centrifugate was transferred to 15 mL centrifuge tube, centrifuged at 1000 rpm for 5 min, and then the supernatant was discarded. After that, 2 mL medium was suspended, and then the cells were counted.

The 96 holes plate was taken and 100 μL PBS solution was added to the outer 36 holes to avoid the edge effect. According to the density of 8000cells/hole, the cells were added into the incubator after diluting and cultured in the incubator. The fresh prepared BLANK-ES, BLANK-bpES, and BLANK-bcES were filtered to remove bacteria, when the cells were fully grown to 80%. The DMEM medium was used to dilute ESs to 2, 4, 8, 16, 32, and 64 times, respectively. Each group was set up with 6 compound holes, and the culture medium was added to each hole with 200 μL medium. After 24 h, the culture medium was sucked. The newly prepared 0.05 mg/mL MTT culture was added, and after 4 h of culture in the incubator, the MTT medium was absorbed and 100 μL DMSO was added to each hole. After the room temperature concussion for 3 min, the OD value of the enzyme labeled instrument was read at 590 nm, and the survival rate of each cell was calculated according to the following formula.

Survival rate (%) = (OD dose/OD control)×100%.

### 3.6. *In vitro* skin permeation studies

The full-thickness skins were obtained from Male Sprague–Dawley rats (weighing 200 ± 20 g, and 5 weeks old). The rats were anesthetized with excess ether inhalation. The abdominal skin was subsequently excised from the animals after removing the abdominal hair, and then the adhering subcutaneous fat or other tissues was removed surgically and washed with PBS.

The mouse skin was kept in the horizontal diffusion cells, and by using saline as accepting liquid, received the fluid volume of 7 mL. The pool has a built-in magnetic stirrer, and was run with a speed of 350 r/min using water bath at 37 °C. 7 mL saline was first added to the receiving pool and then supplied to the pool, balanced for 30 min. Then the liquid in the receiving pool was replaced with fresh saline, the supply chamber was open to the drug, and 7 mL ESs was added to the supply pool, respectively (COL-ES, COL-bpES, COL-bcES). The control group COL36% ethanol aqueous solution (free COL), COL-BO 36% ethanol aqueous solution (free COL-BO, concentration of borneol were the same as that of ethosome, 1.89 mg/mL) at 1, 2, 3, 4, 5, 6, 8, 10, 12, 24, 36, and 48 h, respectively after receiving 1 mL solution were recorded, and was timely supplied with fresh receiving liquid. The absorbed liquid was added to 10 mL after adding 1 mL ethanol. After filtration using microporous filter membrane of 0.22 μm, the continuous filtrate was injected into the high performance liquid chromatograph. The peak area was measured and the corresponding drug concentration was calculated. The cumulative transmission per unit area of Q (μg cm^−2^) and the steady-state transdermal rate Js (μg cm^−2^ h^−1^) were calculated.

After 48 h, the skin was removed, washed with water, cut the site of the medicine, dried the skin surface with ethanol cotton ball, then the skin was cut and extracted suitably. After filtration of the microporous filter membrane of 0.22 μm, the continuous filtrate was injected into the high performance liquid chromatograph to determine the intradermal stagnation amount per unit area. The permeability of colchicine ethanol solution, colchicine borneol ethanol solution, COL-ES, COL-bpES, and COL-bpES five were compared with the internal retention in the skin.

### 3.7. Preparation of ethosomal gels

0.1g carbomer-940 was sprinkled in 10 mL water, maintained at room temperature for overnight, remained for full swelling. Then 0.1 mg/mL sodium hydroxide solution was slowly added, stirred at pH 8, and remained colorless transparent blank gel matrix. The preparation of colchicine alcohol suspension and blank gel matrix were studied in a certain proportion and homogenization, and then the COL plastid gel with COL concentration of 0.2288 mg/mL was obtained.

### 3.8. LC/MS/MS analysis of the drug

The LC/MS/MS system consisted of a solvent delivery pump (1200, Agilent, America), and a triple quadrupole mass spectrometry (6410, Agilent, America). Chromatographic column consisted of Durashell C18 (2.1 × 50 mm, 3 μm); mobile phase of methanol:10 mM ammonium acetate solution (45:55, v/v); flow rate of 0.25 mL/min; sample volume of 20 μL; and column temperature at 20 °C. The ion source involves the electrospray ion source (ESI), positive ion mode, and the injection voltage of 4000 V. The scanning mode involves multiple reaction detection (multi-reactions monitoring, MRM). The quantitative analysis of the ion pairs include Colchicine [M + H]^+^: 400.2–358, and scopolamine hydrobromide [M + H]^+^: 304.2–138. The MS parameters of colchicine and scopolamine hydrobromide were fragmentor (135 V, 95 V), collision energy (30 eV, 22 eV), gas temperature (350 °C, 350 °C), gas flow (8.0 mL min^−1^, 8.0 mL min^−1^) and nebulizer (30 psi, 30 psi), respectively. In all the cases, the flow rate and column temperature were kept at 0.25 mL/min and room temperature, respectively. The linearity, specificity, intra-, and interday variabilities, recovery and matrix effect of these LC/MS/MS methods were also captured and satisfied the quantitative analysis request for the samples.

### 3.9. Pharmacokinetic study of colchicine tablets and ethosomes

A total of 25 SD rats (200 ± 20g, male, Beijing Vital river Co. Ltd.) were randomized into 5 groups. The SD rats in Groups A–E were administered with oral tablets, colchicine ethanol aqueous solution gel, COL-ES gel, COL-bpES gel, and COL-bcES gel (oral tablets, free COL, COL-ES, COL-bpES, COL-bcES), respectively. The concentration of colchicine in the 5 groups was 0.572 mg/kg. The SD rats were fasted for 12 h and fed with drinking water after administration with colchicine. The SD rats in Groups B–E were fed separately in small cages. A volume of 0.5 mL blood sampling was obtained from the inner canthus of SD rats in Group A at 10, 20, 30, 40, 50, 60, 70, 80, 120, and 180 min, respectively after administration of colchicine. In Groups B–E, a volume of 0.5 mL blood sampling was obtained from the inner canthus of SD rats at 0.25, 0.5, 1, 2, 3, 4, 5, 6, 8, 10, 12, 24, 36, and 48 h, respectively after administration with colchicine ethosomes. The blood samplings were then transferred into a 2.5 mL heparinized centrifuge tube and centrifuged at a speed of 3000 r/min for 15 min. The upper layer of the plasma was stored in the centrifuge tube and reserved at −80 °C. After handling the samples, blood concentrations were calculated based on the standard curves and a curve of blood concentration time was determined. Considering that the blood concentration of administration of lower concentration colchicine was greatly infected by the individual differences and artificial errors, the colchicine concentration of the gel was increased to 1.2 mg/kg, and compared the difference of the 4 kinds of gel (*n* = 5).

The data were analyzed by DAS V2.1.1 intelligent analysis model.

### 3.10. Establishment of a model of acute gouty arthritis

Thirty rats were randomly divided into 6 groups, 5 in each group. A group (negative control), B group (model group), model but no drug (model), C group colchicine gel group (free COL), D group colchicine COL-ES gel group (COL-ES), E group COL-bpES gel (COL-bpES), F group COL-bcES gel (COL-bcES), and the ankle circumference was measured in each group. In addition to the normal control group, 0.3 mL 15% sodium urate solution was injected with no.6 injector at the dorsal side of the right ankle joint in the experimental rats. The circumference of the ankle was measured every 6 h with a coiling method, from the initiation of modeling till the administration of the gel.

The administrated gel was about 0.2 cm thick, and the gel was coated above the ankle skin below the right ankle joint at 0.5 cm to prevent the gel from rubbing off by rats. Gel was given once per 12 h, and the gel was removed after each pack of 30 min, and then the gel was wiped out with wet gauze. The total dose of the gel was 1 mg/kg each time. The administration was stopped at 72 h. Then the rats were executed to cut off the knee joint of modeling, and added 4 mL saline to homogenize, and centrifuged at 3000 rpm for 20 min, sucked up the supernatant and stored at −20 °C.

Using TNF-α and PGE2 ELSA kit, the standard curve was produced according to the operation instructions, and the levels of TNF-α and PGE2 in the supernatant of each rat's ankle joint homogenate were measured, respectively.

## 4. Results

### 4.1. Characterization of three kinds of ESs

To better compare the effects of COL-bcES and COL-bpES, the prescription of COL-bpES has been screened in the early stage. The results showed that when the content of borneol was 0.2%, the COL-bpES system was stable and the penetrating effect of the borneol remained certain. Since borneol accounted for 15.6% of the total mass of BO-DOPE, the BO-DOPE mixed with egg yolk lecithin was used to prepare a COL-bcES of 0.2% of the borneol to compare the difference between COL-bcES, and COL-bpES.

**Figure 1.**
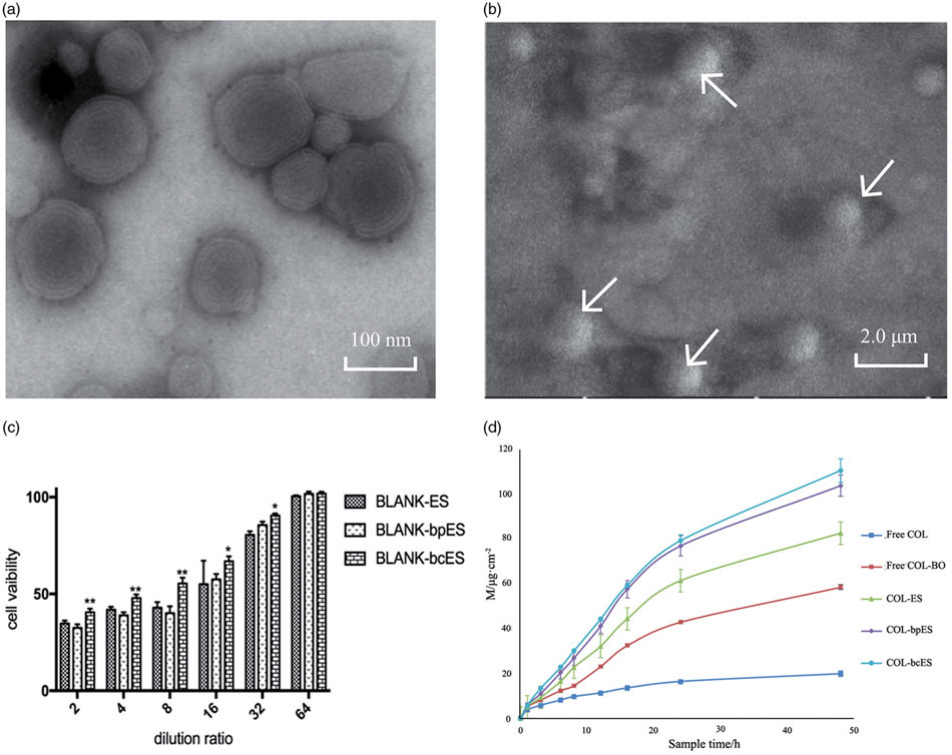
(a) The TEM picture of COL-bcES showing the structure of ethosome as multilayer vesicle; (b) The SEM of COL-bcES showing the stereochemical structure of ethosome as closed sphere; (c) *In vitro* cell viability results of BLANK-ES, BLANK-bpES and BLANK-bcES. The values are expressed as mean ± SD (*n* = 3); *p < .05 and **p < .01 vs BLANK-bpES. There was no significant difference between BLANK-ES and BLANK-bpES; (d) M-t curve of *in vitro* diffusion, the values are presented as mean ± SD (*n* = 3).

The physicochemical properties of COL-ES, COL-bcES, and COL-bpES were showed in Table 1. It can be seen from the diagram that the particle size of ESs prepared by this process was small and the distribution remained narrow. But the stability of COL-bpES was worse than that of COL-ES, and COL-bcES, which was layered after placing at room temperature for 30 d, but COL-ES, and COL-bcES were stable at room temperature after placing at room temperature for 30 d. The TEM and SEM showed that the stereochemical structure of ESs was closed sphere, with multilayer vesicles (Figure 1(a,b)).

**Table 1.** The physicochemical properties of COL-ES, COL-bcES and COL-bpES.

Parameters	COL-ES	COL-bcES	COL-bpES
Size/nm	70.23 ± 2.34	86.33 ± 3.96	104.43 ± 2.81
*Zeta* potential/mV	2.02 ± 0.16	0.73 ± 0.05	1.15 ± 0.05
PDI	0.088 ± 0.033	0.100 ± 0.008	0.045 ± 0.051
Encapsulation efficiency/%	64.96 ± 3.87	62.54 ± 1.49	51.28 ± 0.88

### 4.2. HPLC analysis of the drug

According to the HPLC results, the retention time of COL was 14.6 min, BLANK-ES and COL can be well separated, and have good specificity. The linear results showed that the linearity was good at 2 ∼ 500 μg/mL. The linear equation was A = 73905C + 1485.2 (R^2^=1). The recovery included was 99.91 ± 0.96% (*n* = 3), and the average inter day precision and intraday precision were 0.21% and 0.24% (*n* = 3), respectively.

### 4.3. Cytotoxicity assay

The results of cytotoxicity showed no significant difference between the COL-bpES group and the BLANK-ES group, but when the dilution multiple was lower (2–8 times), the cell activity of the COL-bcES group was significantly different from that of the BLANK-bpES group. But when the dilution multiple was higher (16–32 times), the cell activity of BLANK-bcES group was significantly better than that of BLANK-bpES group (Figure 1(c)). The results showed that the physical modification does not increase the cytotoxicity of the ethosomes, but chemical modification can reduce the cytotoxicity of the whole system, improving the safety of the drug carrying system.

### 4.4. *In vitro* skin permeation studies

The extracorporeal diffusion curve showed that the penetrating rates of borneol ethanol aqueous solution, COL-ES, COL-bpES, and COL-bcES were 2.86, 4.08, 5.13, and 5.47 times, respectively of colchicine ethanol aqueous solution (Figure 1(d)). It can be seen that borneol, even when dissolved in the ethanol aqueous solution, showed a significant penetration promoting effect on drugs. The effect of COL-bcES was stronger than that of COL-bpES.

Other parameters were shown in Table 2. The results showed that the ethosome can increase the accumulation of colchicine in skin, while there was no significant difference between the accumulation amount of COL-bcES and COL-bpES.

**Table 2.** Transdermal permeation parameters.

Sample	Transdermal penetration amountper unit area, μg cm^−2^	Diffusion speed,μg cm^−2^ h^−1^	Enhance ratio	Accumulation of drugs in skin, μg cm^−2^
free COL	20.16 ± 1.21	0.42	1	6.47±1.9
free COL-BO	58.38 ± 1.08	1.2	2.86	8.32 ± 2.3
COL-ES	82.34 ± 5.82	1.72	4.08	13.91 ± 6.9
COL-bpES	103.52 ± 4.80	2.16	5.13	15.77 ± 6.5
COL-bcES	110.28 ± 5.31	2.30	5.47	19.82 ± 7.3

### 4.5. LC/MS/MS analysis of the drug

**Figure 2.**
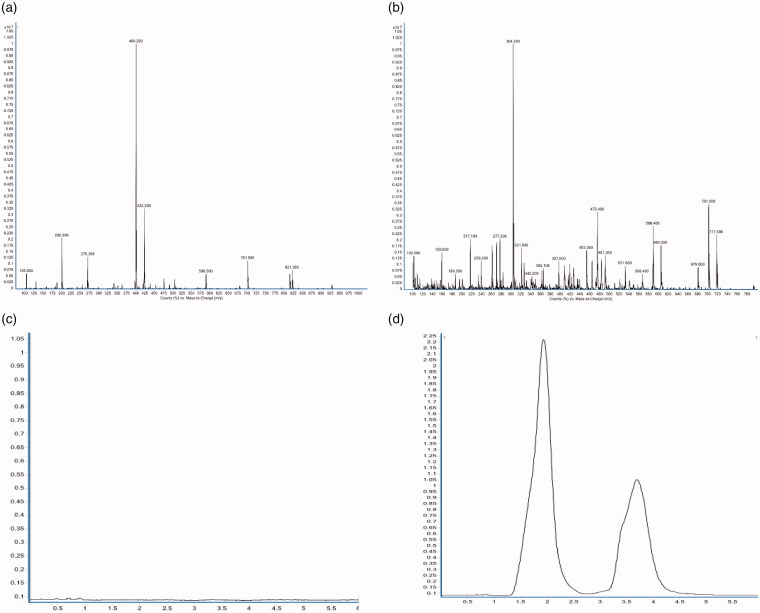
(a) Full-scan mass spectrum of colchicine; (b) Full-scan mass spectrum of scopolamine hydrobromide; (c) MRM chromatogram of blank plasma; (d) MRM chromatogram of colchicine and scopolamine hydrobromide. The rentention time of colchicine and scopolamine hydrobromide was 3.7 min and 1.8 min, respectively.

The full-scan mass spectrum of colchicine and scopolamine hydrobromide and multiple reaction monitoring (MRM) chromatogram of blank plasma, colchicine, and scopolamine hydrobromide were shown in Figure 2(a–d). According to the results, the retention time of colchicine and scopolamine hydrobromide was 3.7 min and 1.8 min, respectively. Colchicine and scopolamine are well separated and had good specificity. The linear results showed that the linearity was good at 0.0 5 ∼ 15 ng/mL. The linear equation was as follows, y = 1.0783x − 0.0755 (R^2^ = 0.99824). The average absolute recovery was 99.24 ± 10.71% (*n* = 9), and the average relative recovery was 90.48 ± 7.19% (*n* = 9). The average inter day precision, intraday precision and the matrix effect were 2.33%, 3.07% (*n* = 3), 106.68 ± 11.49% (*n* = 9), respectively.

### 4.6. Pharmacokinetic study of colchicine tablets and ethosomes

**Figure 3.**
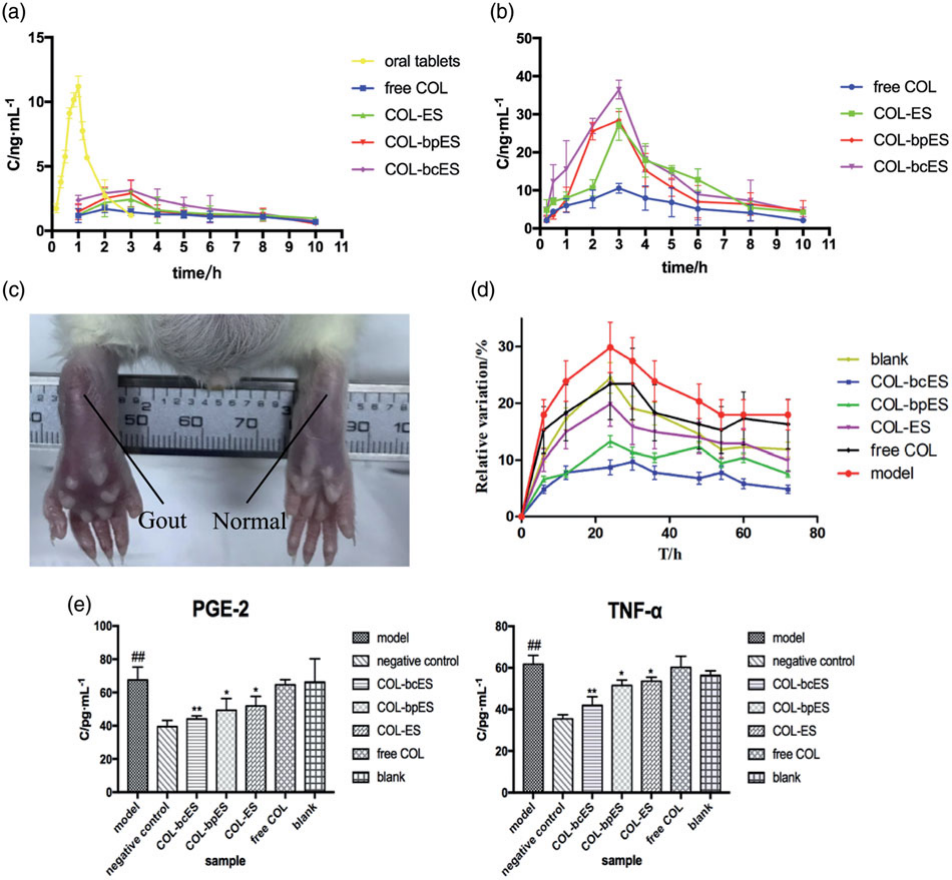
(a) Mean plasma concentration-time curve of dosage of 0.572 mg/kg. The values are expressed as mean ± SD (*n* = 5); (b) Mean plasma concentration-time curve of dosage of 1.2 mg/kg. The values are expressed as mean ± SD (*n* = 5). **p < .01 vs free COL, *p < .05 vs free COL; (c) The swelling degree of rats' joint after molding for 12 h; (d) The perimeter change of rats' joint. The values are expressed as mean ± SD (*n* = 5); (e) The measurement of PGE-2 and TNF-α of rats' joint. The values are expressed as mean ± SD (*n* = 5). ##*p* < .01 vs negative control; **p < .01 vs model, *p < .05 vs model.

The mean plasma concentration-time curve of the 4 groups of transdermal doses of 0.57 mg/kg and the plasma concentration-time curve of the oral group were shown in Figure 3(a) and the mean plasma concentration-time curve of the 4 groups of transdermal doses of 1.2 mg/kg were shown in Figure 3(b). According to the minimum principle of AIC, the best oral model was two compartment model, while the transdermal drug delivery group was calculated by a non-compartment model. The main pharmacokinetic parameters are shown in Tables 3 and 4. According to the results of pharmacokinetics, transdermal delivery can produce an obvious sustained release effect. The time curve of oral administration of common colchicine tablets showed that its concentration was fluctuated *in vivo* and reached *C*_max_ at 1 h after administration. The blood concentration profile with two doses had similar tendency. The transdermal delivery of *T*_max_ was longer than 2.5 h, delayed the peak time, and stabilized the blood concentration. According to the pharmacokinetic parameters, the order of *AUC*_(0−∞)_, *AUC*_(0−t)_ and *C*_max_ were all COL-bcES > COL-bpES > COL-ES > free COL, and showed no significant differences between free COL and COL-ES, COL-bpES, COL-bcES. But there were no significant differences of *MRT*_(0−t)_, *MRT*_(0−∞)_ and *T*_max_. The results of mean resident time (MRT) and *T*_max_ showed that ethosome cannot change the elimination of COL and drug concentration reached the peak in similar time. The significant differences of AUC and *C*_max_ between different groups indicated that different ethosomes can enhance transdermal permeation rate, which was beneficial of improvement of therapeutic effect.

**Table 3.** Main pharmacokinetic parameters with dosage of 0.572 mg/kg.

Parameters	Unit	Oral tablets	Free COL	COL-ES	COL-bpES	COL-bcES
*AUC*_(0−t)_	ng/mL	11.63 ± 2.33	11.37 ± 0.14	14.22 ± 4.39	14.56 ± 2.08**	18.74 ± 2.56**
*AUC*_(0−∞)_	ng/mL	13.11 ± 1.88	18.78 ± 5.01	30.37 ± 13.38	17.90 ± 0.80	22.39 ± 3.18
*MRT*_(0−t)_	h	1.02 ± 0.15	4.79 ± 0.39	4.71 ± 0.08	4.41 ± 0.45	4.30 ± 0.24*
*MRT*_(0−∞)_	h	1.21 ± 0.14	11.19 ± 4.80	17.79 ± 13.28	6.73 ± 1.51	6.00 ± 0.71*
*T*_max_	h	1.00 ± 0.00	3.40 ± 1.95	2.80 ± 0.45	2.40 ± 0.89	3.40 ± 1.14
*C*_max_	ng/mL	11.19 ± 0.80	1.74 ± 0.22	2.74 ± 0.71	3.07 ± 0.82**	3.56 ± 0.53**

The values are expressed as mean  ±  s (*n* = 5). **p < .01 vs free COL; *p < .05 vs free COL.

**Table 4.** Main pharmacokinetic parameters with dosage of 1.2 mg/kg.

Parameters	Unit	Free COL	COL-ES	COL-bpES	COL-bcES
*AUC*_(0−t)_	ng/mL	57.93 ± 10.29	115.64 ± 15.66**	115.84 ± 5.38**	145.65 ± 30.29**
*AUC*_(0−∞)_	ng/mL	82.30 ± 35.86	130.70 ± 16.62*	147.91 ± 27.26*	161.69 ± 38.29**##
*MRT*_(0−t)_	h	4.29 ± 0.41	4.38 ± 0.15	4.07 ± 0.24	3.81 ± 0.31
*MRT*_(0−∞)_	h	9.52 ± 8.44	5.46 ± 0.27	6.27 ± 1.93	4.75 ± 0.90
*T*_max_	h	3.60 ± 0.89	3.00 ± 0.00	2.60 ± 0.55	3.00 ± 0.00
*C*_max_	ng/mL	11.03 ± 0.78	27.32 ± 4.19**	28.98 ± 1.73**	36.48 ± 2.45**

The values are expressed as mean  ±  s (*n* = 5). ##*p* < .01 vs COL-bpES; **p < .01 vs free COL; *p < .05 vs free COL.

### 4.8. Establishment of acute gouty arthritis model

The joint of the rat at 12 h after the establishment of the model was shown in Figure 3(c). It can be seen that the joints on the left showed obvious swelling compared with the joints on the right side.

From the change of circumference, it can be seen that the joint circumference was increased most obviously in the model group without drug administration, and the swelling degree reached highest at 24 h. The growth changes of COL-bcES and COL-bpES were shown in Figure 3(d). This indicated that after administration of the drug, the drug enters the joint through the skin and inhibits the occurrence of inflammation. At the end of administration, the results of inflammatory factors (TNF-α and PGE2) were shown in Figure 3(e).

According to the results of inflammatory factors, the inflammatory factors in the model group and the control group were significantly different. Compared with the model group, there was a significant difference between COL-bcES and the model group. There was a significant difference in the COL-bpES and COL-ES groups compared with the model group, but colchicine ethanol solution group showed no significant difference with the model group. This proved that the ethosome increased the amount of colchicine penetration into the joint cavity, thus inhibiting the formation of inflammatory factors and acute gout. This involves certain therapeutic effect.

The results of pharmacokinetics and treatment study showed that COL-bcES had obvious advantages than COL-bpES. And why these happened has been clearly explained in literatures so far. It was assumed that borneol of COL-bcES had more stable distribution in surface of nanocarrier when ES passed through skin. For extreme lipophicity of borneol, it was easy to be encapsulated into the lipid core of COL-bpES so that littler borneol can take effect. Therefore, COL-bcES can overcome effectively weakness to obtain better utilization of borneol but assumption need further research data to be proved.

## 5. Conclusion

On the basis of early laboratory research, the synthetic BO-DOPE was further purified in this study and colchicine was selected as a model drug for studying transdermal drug delivery system, and its adaptation to acute gout attack was selected as the research object. COL-ES, COL-bpES, and COL-bcES were constructed and compared their size, Zeta potential, cytotoxicity, *in vitro* diffusion, elementary pharmacokinetics, and pharmacodynamics. Considering that ethanol and borneol in COL-bES system are traditional percutaneous penetration enhancers, the effect of COL-bcES and COL-bpES system was determined. The experiment selected ethanol solution of colchicine and colchicine mixed borneol as controls. The results suggested that the ethosome demonstrated a better transdermal effect, and when compared with the physical modification system showed better stability, less cytotoxicity, higher plasma concentrations, and better anti-inflammatory effects of chemical modification system, providing a good delivery system for the treatment of small molecule inflammatory drugs.
